# 

**Published:** 1845-10

**Authors:** 


CONTENTS:

Cases of Surgery. Compression of the Brain by Effusion of Blood—Bony Concretion of the Knee Joint—Tracheotomy. By DANIEL BRAINARD, M.D., Professor of Surgery in Rush Medical College, - - - 97

BIBLIOGRAPHICAL NOTICES.

Minutes of the Organization, Constitution, and Ethics, of the Union Medical Society of Northern Indiana, - - - 101
Medical Journals, - - 102

MEDICAL INTELLIGENCE.

National Medical Convention, - 104

Salacine, - - - 104

PRACTICAL MEDICINE, &c.

On the Use of Ioduret of Potassium in Syphiltic Affections, - 105
On the Dropsy which follows Scarlatina, - - - 106
On the Remedial Efficacy of Ox-Gall. - - - - 107
Valerianate of Quinia, - 108
Of the Use of Stimulants in Inflammatory Diseases of the Lungs in Children, - - - 109
Cyanosis of Infants, - - 110
Ipecacuanha, in Emetic Doses, as a Powerful Restorative in some Cases of Exhaustion and Sinking, - - - - 111
Vinegar in Cases of Narcotic Poisoning, - - - 112
Hooping Cough, - - 112

